# Real-World Efficacy of Beclomethasone Dipropionate/Formoterol Fumarate/Glycopyrronium on Diaphragmatic Workload Assessed by Ultrasound and Lung Function in Patients with Uncontrolled Asthma

**DOI:** 10.3390/arm93050040

**Published:** 2025-10-01

**Authors:** Antonio Maiorano, Anna Ferrante Bannera, Chiara Lupia, Daniela Pastore, Emanuela Chiarella, Giovanna Lucia Piazzetta, Angelantonio Maglio, Alessandro Vatrella, Girolamo Pelaia, Corrado Pelaia

**Affiliations:** 1 Department of Health Sciences, Magna Graecia University, Viale Europa—Località Germaneto, 88100 Catanzaro, Italy; antoniomaiorano95@gmail.com (A.M.); annaferrantebannera@gmail.com (A.F.B.); chiara.lupia@hotmail.it (C.L.); danielapastore11@gmail.com (D.P.); pelaia@unicz.it (G.P.); 2 Department of Medical and Surgical Sciences, Magna Graecia University, Viale Europa—Località Germaneto, 88100 Catanzaro, Italy; emanuelachiarella@unicz.it (E.C.); giovannapiazzetta@hotmail.it (G.L.P.); 3 Department of Medicine, Surgery and Dentistry, University of Salerno, 84084 Salerno, Italy; amaglio@unisa.it (A.M.); avatrella@unisa.it (A.V.)

**Keywords:** uncontrolled asthma, inhaled therapy, lung function, small airway disease, diaphragmatic function, thoracic ultrasound

## Abstract

**Highlights:**

**What are the main findings?**

- Triple inhaled therapy with beclomethasone dipropionate/formoterol fumarate/glycopyrronium (BDP/FF/G) significantly improved airflow, reduced residual volume, and enhanced symptom control in patients with uncontrolled asthma.
- Diaphragmatic ultrasound showed a marked reduction in diaphragmatic thickening fraction, indicating decreased respiratory workload.

**What is the implication of the main finding?**

- These results suggest that triple inhaled therapy may not only improve lung function and symptoms but also reduce respiratory muscle strain in real-world settings.
- Diaphragmatic ultrasound could represent a novel, non-invasive biomarker to monitor treatment response in uncontrolled asthma.

**Abstract:**

**Background:** Uncontrolled asthma remains a significant clinical challenge, often linked to impaired lung function and increased diaphragmatic workload. Recent studies have shown promising results using a triple inhaled therapy comprising beclomethasone dipropionate/formoterol fumarate/glycopyrronium (BDP/FF/G). This study assessed the real-world efficacy of BDP/FF/G on lung function and diaphragmatic workload in patients with uncontrolled asthma. **Methods:** A prospective observational study enrolled 21 adult patients diagnosed with uncontrolled asthma despite high-dose ICS/LABA therapy. Patients underwent lung function tests and right diaphragmatic ultrasound assessments at baseline and after three months of treatment with BDP/FF/G (172/5/9 mcg, administered as two inhalations every 12 h). **Results:** After three months, significant improvements were observed in FEV_1_ (from 57.75 ± 12.30% to 75.10 ± 18.94%, *p* < 0.001) and FEF_25–75_ (from 47.80 ± 19.23% to 75.10 ± 36.06%, *p* < 0.001). Additionally, during the same period, we recorded significant reductions in residual volume (from 130.10 ± 28.20% to 92.55 ± 21.18%, *p* < 0.001) and total airway resistance (R_tot_) (from 164.60 ± 83.21% to 140.70 ± 83.25%, *p* < 0.05). The mean asthma control test (ACT) score increased by 5.6 points (*p* < 0.001), surpassing the established minimal clinically important difference (MCID) of 3 points and raising the cohort mean above the well-controlled threshold. The right diaphragmatic workload was significantly decreased, as shown by a reduction in thickening fraction (TF) (from 63.86 ± 17.67% to 40.29 ± 16.65%, *p* < 0.01). Correlation analysis indicated significant associations between diaphragmatic function and some lung function parameters (FEV_1_, FEF_25–75_, and R_tot_). **Conclusions:** In this real-world pilot, triple BDP/FF/G was linked to improvements in airflow, hyperinflation, symptoms, and a reduction in diaphragmatic thickening fraction, indicating potential physiological benefit. Due to the small sample size, single-centre design, and 3-month follow-up, these results should be viewed as hypothesis-generating and need to be confirmed in larger, controlled, multicentre studies with longer follow-up.

## 1. Introduction

Asthma is a chronic inflammatory condition that affects the airways, typically causing wheezing, reversible airflow obstruction, and bronchial hyperresponsiveness. The global impact of asthma is significant, with over 300 million people worldwide affected [1]. While airway inflammation is a key feature of asthma, the disease presents in various ways, with distinct subtypes known as phenotypes. These phenotypes are driven by complex cellular and molecular mechanisms, referred to as endotypes [2]. These interactions describe type 2 (T2)-high or T2-low asthma. T2-high eosinophilic allergic disease develops as a result of crosstalk between adaptive and innate immune responses, driven by T helper 2 cells (Th2), group 2 innate lymphoid cells (ILC2), interleukin (IL)-13, IL-5, and IL-4. Airborne pollutants, respiratory viruses, cigarette smoking, and aeroallergens amplify the action of these interleukins. These agents induce bronchial epithelial cells to produce IL-25, thymic stromal lymphopoietin (TSLP), and IL-33. T2-low asthma is characterised by airway neutrophilia. T helper 17 cells (Th17), group 3 innate lymphoid cells (ILC3), IL-17A, IL-17F, IL-6, IL-1β, IL-23, and TGF-β are fundamental for its neutrophilic pathogenesis. Diesel exhaust particles and cigarette smoke could promote airway neutrophilia.

A mixed eosinophilic/neutrophilic inflammatory phenotype may be associated with severe asthma, where IL-4 and IL-17A are produced at the same time [3]. In medical practice, inhaled therapy is recommended as a pharmaceutical treatment for patients with asthma. These treatments aim to minimise both the symptom burden and the risk of adverse asthma outcomes, as well as exacerbations, persistent airflow limitation, and medication side effects [4]. According to the Global Initiative for Asthma (GINA) guidelines, asthma treatment typically begins with an inhaled corticosteroid (ICS) and a long-acting beta agonist (LABA) and may be further advanced by adding a long-acting muscarinic antagonist (LAMA) if necessary [5]. Recently, two significant phase III trials (TRIMARAN and TRIGGER) have demonstrated the efficacy of single inhaled triple therapy with beclomethasone dipropionate/formoterol fumarate/glycopyrronium (BDP/FF/G) in patients with uncontrolled asthma. Notably, it has been shown that a triple inhaled combination is superior to dual therapy with a medium or high dose of ICS/LABA in terms of improving lung function and preventing asthma exacerbations [6]. It is an extra-fine formulation with a median aerodynamic diameter (MMAD) < 2 μm [7] that can reach both large bronchi and small bronchioles, up to peripheral airways < 2 mm in diameter [8]. Asthmatic patients with significant dysfunction of the small airways are inclined to have worse quality of life and asthma control, increasing exacerbation risk [9]. In the literature, there is limited evidence regarding the application of thoracic ultrasound (TUS) in adult asthmatic patients; however, its use is increasing, particularly in the evaluation of the right heart, liver, and pleural effusion [10,11,12,13]. In particular, hyperinflation and altered inspiratory mechanics increase the workload on the diaphragm in obstructive lung disease. In chronic obstructive pulmonary disease (COPD), diaphragm ultrasound—especially the thickening fraction—monitors disease severity, hyperinflation, and symptoms, and responds to therapeutic deflation [14]. Since small-airway dysfunction and air trapping also occur in asthma, we hypothesised that the diaphragmatic thickening fraction could act as a non-invasive marker of inspiratory workload and might improve alongside spirometric indices and symptoms when hyperinflation is reduced. Because single-inhaler triple therapy BDP/FF/G can decrease airway resistance and air trapping [15], we proposed that clinical and spirometric improvements would coincide with a reduction in diaphragmatic thickening fraction, indicating lower inspiratory effort. Moreover, an improvement in right diaphragmatic workload after three months of triple inhaled therapy with BDP/FF/G was previously observed in an adult patient with uncontrolled asthma [16]. To our knowledge, no observational studies have evaluated the efficacy of BDP/FF/G in adult asthmatic patients with uncontrolled asthma regarding right diaphragmatic workload and lung function. Based on this evidence, our primary objective was to evaluate the improvement in real-life lung function tests after three months of therapy. The second aim was to investigate whether the improvement in lung function test results was related to a reduction in right diaphragmatic effort, as assessed by ultrasound evaluation.

## 2. Materials and Methods

### 2.1. Study Setting and Lung Function Tests

In this pilot prospective cohort study, twenty-one patients (eight males and thirteen females) with uncontrolled asthma during single inhaler therapy ICS/LABA were evaluated at the Respiratory Unit of “Magna Graecia” University Hospital of Catanzaro, Italy. Uncontrolled asthma was diagnosed according to GINA guidelines [5]. Lung function tests were conducted following the guidelines of the American Thoracic Society (ATS) and European Respiratory Society (ERS) [17,18], utilising the Master Screen Pulmonary Function Testing System and the Master Screen Body (Jaeger, Germany). Asthma was defined according to GINA guidelines [5]. Specifically, all patients underwent lung function tests with bronchodilator reversibility testing, demonstrating a forced expiratory volume in one second (FEV_1_) improvement of more than 200 mL in absolute value and 12% in percentage value after inhaling 400 micrograms of salbutamol. At baseline, all patients, despite taking a high dose of ICS/LABA and demonstrating good inhalation technique, experienced frequent exacerbations characterised by cough, shortness of breath, wheeze, chest tightness, and exercise limitation, with an asthma control test (ACT) score of less than twenty. Any of these criteria were used to determine the presence of underlying T2 inflammation (T2-high): (i) at least two FeNO assessments showing 25 ppb or higher; (ii) at least two blood eosinophil measurements with counts of 150 cells/μL or more; (iii) clinically significant allergies with linked sensitization. BDP/FF/G 172/5/9 mcg was prescribed according to the eligibility indications and was administered at a dosage of two inhalations every 12 h. FEV_1_, forced vital capacity (FVC), forced expiratory flow at 25–75% of FVC (FEF_25–75_), residual volume (RV), total airway resistance (R_tot_), and effective resistance (R_eff_) were evaluated at baseline and after three months of triple inhaled therapy with BDP/FF/G. We also monitored drug safety and tolerability during monthly visits, checking for infections or any health decline. The lack of a comparator precludes causal inference and was prespecified as a design limitation to be addressed in a subsequent, controlled study (randomised or matched cohort). This observational study followed Good Clinical Practice (GCP) standards and the principles of the Declaration of Helsinki. It received approval from the Ethics Committee of the Calabria Region, Italy (Catanzaro, Italy; authorisation number 9, 11 January 2024). All participants provided written informed consent.

### 2.2. Diaphragmatic Ultrasound Protocol

Diaphragmatic ultrasound was carried out according to a previous case report conducted by our research group [16]. Briefly, we performed a baseline right diaphragmatic ultrasound, conducted by a pulmonologist with extensive experience in this field. The ultrasound evaluator was blinded to the clinical data. Measurements were taken during spontaneous and resting tidal breathing in both seated and supine positions. A 3.5–5 MHz convex ultrasound probe in brightness mode (B-mode) was used for thickness, and a 7–15 MHz linear probe in B-mode and motion mode (M-mode) was applied for shift, in abdominal and musculoskeletal settings, respectively (Esaote MyLab XPro 30, Esaote S.p.A., Genoa, Italy). An echographic window was identified for the right hemidiaphragm starting from the liver. While lying down, the convex probe was placed between the midclavicular and anterior axillary lines, using B-mode imaging to examine the right hemidiaphragm. During inspiration, the diaphragm contracted and moved towards the transducer. This motion was recorded with M-mode, measuring the vertical height from baseline to the point of maximum inspiration on a frozen image. At least three consecutive respiratory cycles were analysed to ensure measurement accuracy. During the seated position, the right diaphragmatic thickness was assessed using a linear probe settled in B-mode. It was placed below the phrenicocostal sinus, near the mid-axillary line. The probe was positioned below the phrenicocostal sinus, near the mid-axillary line at the eighth intercostal space, where the diaphragm appeared as a structure between two parallel echogenic lines. The left hemidiaphragm was not evaluated due to variable gastric positioning and limited time. This decision improves within-subject consistency but restricts bilateral applicability. The measurements were taken at the end of expiration and inspiration during quiet breathing at tidal volume, as well as at the end of inspiration following deep breathing, across three different breathing cycles. Normal ranges were based on data from Boussuges et al. [19,20] and Zambon et al. [21]. After three months of triple inhaled therapy with BDP/FF/G, a subsequent right diaphragmatic assessment was conducted.

### 2.3. Statistical Analysis

The mean ± standard deviation (SD) was used for normally distributed data, while the median value with interquartile range (IQR) was used for data distributions with skewed distributions. Based on the normality assumption, either parametric or non-parametric tests were selected. Data normality was evaluated with the Anderson-Darling and Kolmogorov–Smirnov tests. When appropriate, the Wilcoxon signed-rank test and paired *t*-test were performed to compare variables. A Pearson correlation matrix was used to analyse the linear relationships among the tested groups. A *p*-value of less than 0.05 (two-tailed) was deemed statistically significant. Statistical analyses and figures were generated using Prism Version 10.3.0 software (GraphPad Software Inc., San Diego, CA, USA).

## 3. Results

We have enrolled 21 patients (8 males and 13 females), with a mean age of 66.10 ± 9.66 years, and a body mass index (BMI) of 27.50 (21.00–30.50) kg/m^2^. The mean baseline FEV_1_ value was 57.75 ± 12.30%, and the mean baseline RV was 130.10 ± 28.20%. The main baseline patient characteristics are summarised in Table 1.

**Figure 1:**
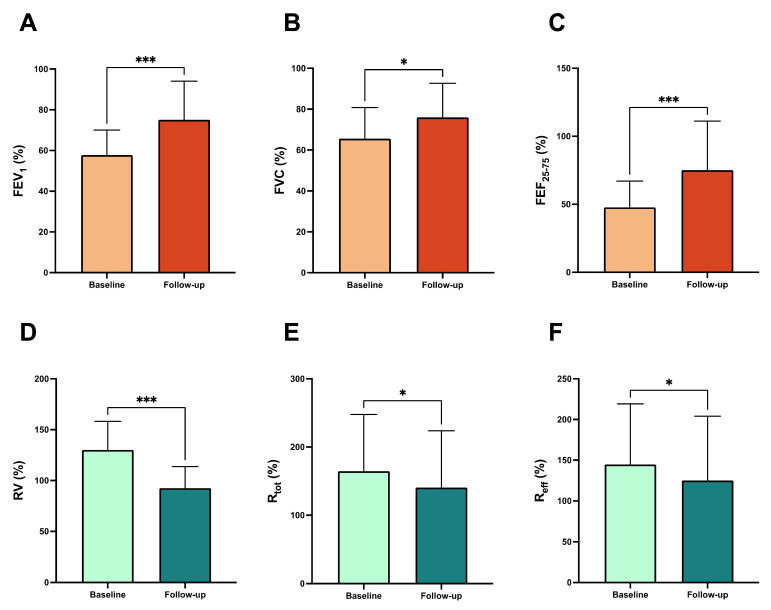
Efficacy of BDP/FF/G regarding lung function outcomes, FEV_1_ (**A**), FVC (**B**), FEF_25–75_ (**C**), RV (**D**), R_tot_ (**E**), and R_eff_ (**F**). * *p* < 0.05, *** *p* < 0.001. Abbreviations: FEV_1_, forced expiratory volume in one second; FVC, forced vital capacity; FEF_25–75_, forced mid-expiratory flow between 25% and 75% of forced vital capacity; RV, residual volume; R_tot_, total resistance; R_eff_, effective resistance.

**Figure 2:**
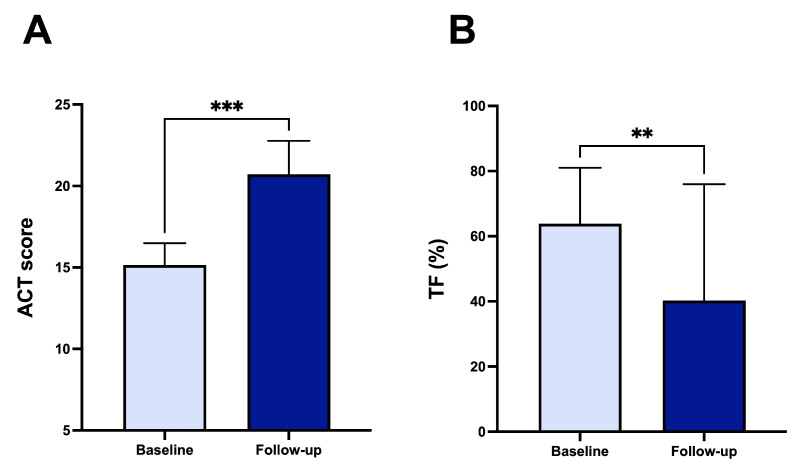
Efficacy of BDP/FF/G regarding ACT score (**A**) and TF (**B**). ** *p* < 0.01, *** *p* < 0.001. Abbreviations: ACT, asthma control test; TF, diaphragmatic thickening fraction.

**Figure 3:**
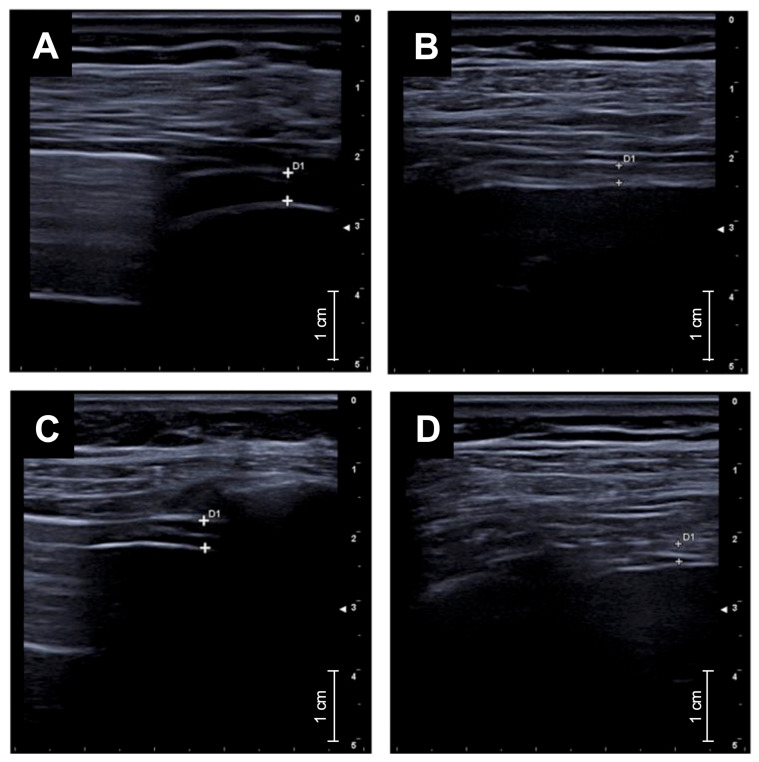
Diaphragmatic ultrasound images showing inspiratory and expiratory thickness at baseline (**A**,**B**) and after three months of triple inhaled therapy BDP/FF/G (**C**,**D**).

After three months of triple inhaled therapy with BDP/FF/G, FEV_1_ increased from 1.49 ± 0.43 L to 1.91 ± 0.59 L (*p* < 0.001) and from 57.75 ± 12.30% to 75.10 ± 18.94% (*p* < 0.001) (Figure 1A). During the same period, FVC changed from 2.15 ± 0.55 L to 2.45 ± 0.69 L (*p* < 0.05) and from 65.60 ± 15.17% to 76.05 ± 16.59% (*p* < 0.05) (Figure 1B). FEF_25–75_ improved from 1.09 ± 0.55 L/s to 1.69 ± 0.95 L/s (*p* < 0.001) and from 47.80 ± 19.23% to 75.10 ± 36.06% (*p* < 0.001) (Figure 1C). Moreover, RV decreased from 2.05 ± 0.69 L to 1.68 ± 0.71 L (*p* < 0.05), and from 130.10 ± 28.20% to 92.55 ± 21.18% (*p* < 0.001) (Figure 1D). Regarding the effects on airway resistance, triple inhaled therapy reduced both R_tot_, from 0.49 ± 0.23 kPa*s/L to 0.41 ± 0.21 kPa*s/L (*p* < 0.05) and from 164.60 ± 83.21% to 140.70 ± 83.25% (*p* < 0.05) (Figure 1E). R_eff_ changed from 0.43 ± 0.20 kPa*s to 0.36 ± 0.21 kPa*s (*p* < 0.05) and from 144.7 ± 74.50% to 125.1 ± 78.99% (*p* < 0.05) (Figure 1F). We also considered the clinical impact of triple combined inhaled therapy, as measured by ACT score, revealing an improvement in its value from 15.14 ± 1.34 to 20.71 ± 2.06 (*p* < 0.001) after three months of treatment (Figure 2A). Moreover, we have analysed the efficacy of BDP/FF/G on right diaphragmatic workload using ultrasound, finding a reduction in diaphragmatic thickening fraction (TF), from 63.86 ± 17.67% to 40.29 ± 16.65% (*p* < 0.01) (Figure 2B). Figure 3 shows diaphragmatic ultrasound images documenting inspiratory and expiratory thickness at baseline (Figure 3A,B) and after three months of triple inhaled therapy BDP/FF/G (Figure 3C,D), respectively.

**Figure 4:**
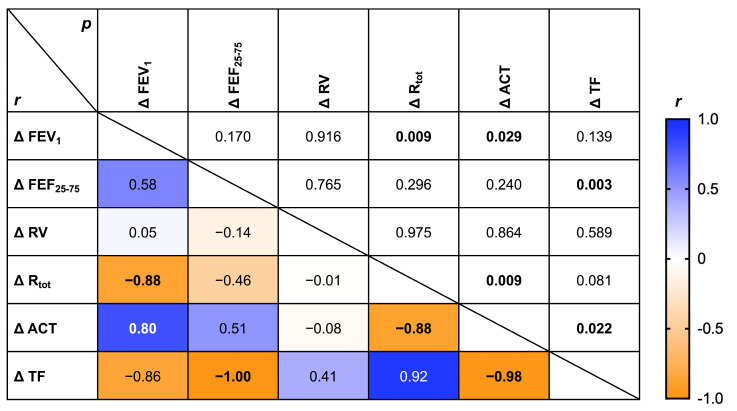
Correlation matrix assessing relationships among changes in functional, clinical, and diaphragmatic parameters after BDP/FF/G inhaled therapy.

In addition, by running correlation matrix, statistically significant associations emerged between increase in FEV_1_ and decrease in R_tot_ (*r* = −0.88, *p* < 0.001), increase in FEV_1_ and increase in ACT (*r* = 0.80, *p* < 0.05), decrease in R_tot_ and increase in ACT (*r* = −0.88, *p* < 0.01), rise in FEF_25–75_ and decrease in TF (*r* = −0.88, *p* < 0.01), increase in FEV_1_ and decrease in TF (*r* = −0.86, *p* < 0.05) (Figure 4).

Furthermore, all patients adhered optimally to inhaled BDP/FF/G treatment, with no side effects or adverse reactions reported during the monthly visits. All enrolled participants completed the month-3 visit and were included in the analysis (retention 100%; no dropouts). No discontinuations occurred due to adverse events.

## 4. Discussion

To our knowledge, this is the first observational study to demonstrate real-life improvement in lung function and its association with a reduction in diaphragmatic workload after three months of triple inhaled therapy with BDP/FF/G. Asthma can result in changes to the airway structure, including neo-angiogenesis, subepithelial fibrosis, smooth muscle thickening, and goblet cell metaplasia/hyperplasia [22,23]. The efficacy of BDP/FF/G in improving lung function tests has been proven, with a positive effect on severe exacerbations and some benefit in terms of symptom control in adult patients with uncontrolled asthma [24]. However, there are no real-life studies that attest to its effectiveness in improving lung and diaphragmatic function. In asthmatic patients, particularly in obese individuals, airway inflammation can lead to expiratory airflow limitation and dynamic hyperinflation, thereby reducing fitness for activities of daily living [25]. This condition may lead to an increase in diaphragmatic workload, as seen in patients with COPD due to their pulmonary hyperinflation. Respiratory muscle fibres show several impairments in cellular structures, which are proportional to the severity of the disease. The diaphragm can express adaptive changes in response to the chronic mechanical load imposed by the disease, with a “fragile balance” between respiratory muscle overload and respiratory muscle adaptations [16,26,27].

**Figure 5:**
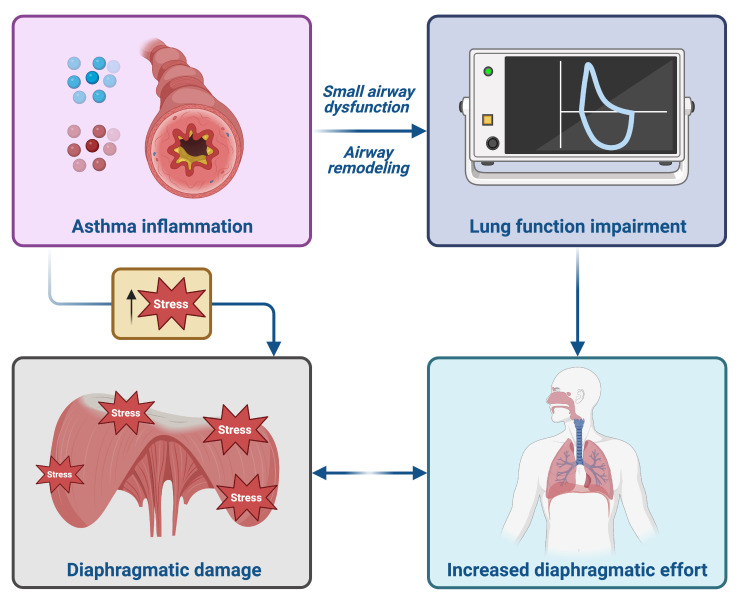
Pathogenic hypothesis of increased right diaphragmatic effort in adult patients with uncontrolled asthma. This original figure was created by the authors using “BioRender.com”, https://www.biorender.com (accessed on 5 August 2025).

In this way, the application of diaphragmatic ultrasound in patients with COPD is well established [15], but its potential role in adult asthmatic patients remains undefined. Starting from this evidence, we have enrolled twenty-one adult patients with uncontrolled asthma, who were taking high doses of ICS/LABA with no benefit. Baseline lung function tests and ACT score were obtained, demonstrating severe reversible obstruction and poor control of asthma symptoms, with a score less than 20. We also performed baseline right diaphragmatic ultrasound evaluation, showing high diaphragmatic effort, defined by increased TF. This diaphragmatic ultrasound evidence differs from that of Portacci et al. They evaluated diaphragmatic function in adult asthmatic patients, pointing out a dysfunction of this muscle, with a reduced TF compared to healthy controls [28]. This discrepancy with our study could be due to the different sample of patients evaluated. We have enrolled adult patients with uncontrolled asthma, characterised by worse spirometric values, hyperinflation, and a consequent increase in TF, due to high diaphragmatic workload. Therefore, we decided to change inhaled therapy to a high dose of BDP/FF/G, two inhalations every 12 h. After three months, follow-up lung function tests were performed, revealing improvements in FEV_1_, FEF_25–75_, RV, and R_tot_, along with an improvement in the ACT score. These results could be explained by the distribution of BDP/FF/G. Both large bronchi and small bronchioles experience improved deposition of the extrafine formulation in the small airways [29]. The anti-inflammatory effects at the bronchiolar level from extra-fine beclomethasone dipropionate could be amplified by combining it with extra-fine formoterol fumarate and glycopyrronium, leading to relaxation of the small airway smooth muscle. Such a condition could induce bronchodilation, improve expiratory airflow, and thereby reduce dynamic lung hyperinflation [30]. Consequently, these effects might decrease the diaphragmatic workload. In fact, in hyperinflated patients, the diaphragm has a higher workload due to increased mechanical loads and airflow limitation. This led to adaptive changes in response to the chronic mechanical load imposed [25] (Figure 5).

After three months of triple inhaled therapy with BDP/FF/G, the improvement in FEV_1_, FVC, and RV was associated with a decrease in right diaphragmatic workload. In particular, we have found a statistically significant correlation between the improvement of TF and FEF_25–75_, which contrasts with the evidence presented by Eysa et al. [31]. In our cohort, diaphragmatic TF decreased alongside improvements in FEV_1_ and FEF_25–75_, whereas Eysa et al. reported a positive correlation between TF and FEF_25–75_. Several factors likely explain this divergence. Firstly, baseline severity and hyperinflation: a significant proportion of our patients were GINA Step 4–5 with evident deflation during follow-up; in this context, treatment reduces inspiratory load so TF falls at similar tidal volumes, even as flows improve. In milder disease with limited air trapping—as seen in Eysa et al.—TF may instead reflect contractile recruitment or reserve, resulting in a positive association with flow. Moreover, this result is not coherent with a hypothetical state of remodelling of the bronchial walls and small airways, which should cause an increase in TF, which cannot therefore be associated with an increase in FEF_25–75_. Moreover, a statistically significant correlation exists between improvements in TF scores and ACT scores, as well as between TF and FEV_1_. These results demonstrate the real-life efficacy of BDP/FF/G on lung function and represent, for the first time, a potential novel approach for ultrasound follow-up of adult patients with uncontrolled asthma during this triple inhaled therapy. However, several limitations should be acknowledged. First, the small sample size (n = 21) reduces statistical power and raises the risk of both type I and type II errors. For this reason, all correlation findings are exploratory and derived from a small pilot cohort; they should be replicated using standardised ultrasound protocols and a priori statistical plans in multicentre studies. Second, the short follow-up period of three months limits the ability to draw conclusions about the long-term effects of triple therapy on diaphragmatic function and asthma control. Third, the single-centre design may restrict the generalisability to broader patient groups. Fourth, the lack of a control group receiving alternative treatments (e.g., high-dose ICS/LABA or ICS/LABA/LAMA via separate inhalers) prevents direct comparative analysis. The pilot design and modest sample size limit precision and prevent definitive subgroup analyses. Importantly, the absence of a concurrent control group restricts causal inference and may inflate effect estimates due to regression to the mean or co-interventions. Future research should employ a controlled, adequately powered design—preferably randomised or a matched cohort—with blinded dual-rater ultrasound analysis to verify whether reductions in hyperinflation and diaphragmatic TF are due to BDP/FF/G. Moreover, although we outlined mechanistic distinctions between T2-high and T2-low asthma, the present cohort was not powered or pre-specified for endotype-based analyses. Baseline endotypes are reported, but no formal stratified comparisons were performed due to the small sample size.

Although there are some limitations, this study demonstrates that thoracic ultrasound can be a feasible method to evaluate diaphragmatic function in asthma. It also offers initial evidence that triple therapy might improve lung function and reduce respiratory muscle workload in real-world settings. Diaphragm ultrasound was feasible and physiologically consistent with the deflation we observed; however, these findings are hypothesis-generating. Ultrasound assessments were limited to the right hemidiaphragm to maximise the acoustic window and ensure standardisation; left-sided measurements were not performed, restricting bilateral evaluation comparison. Prospective validation in larger, controlled cohorts—with standardised acquisition (zone of apposition, tidal breathing, left hemidiaphragm assessment), blinded dual-rater readings, and pre-specified minimal detectable change thresholds—is necessary before clinical implementation. Moreover, the 3-month follow-up limits conclusions on long-term efficacy and durability. Future studies should evaluate whether improvements in airflow, hyperinflation, symptoms, and TF are maintained at 6–12 months, and whether they lead to fewer severe exacerbations, lower oral corticosteroid use, and improved health status and quality of life measures. Therefore, larger, controlled studies with longer follow-up periods are necessary to confirm these results and to determine if diaphragmatic ultrasound can be incorporated into regular asthma monitoring.

## 5. Conclusions

In a small, single-centre pilot cohort, initiation of BDP/FF/G was linked with improvements in spirometry, residual volume, patient-reported control, and a decrease in diaphragmatic thickening fraction, a pattern consistent with reduced inspiratory workload. While promising, these findings should be interpreted with caution due to the modest sample size, lack of a concurrent control group, and short (3-month) follow-up. Validation in larger, multicentre, controlled cohorts—preferably randomised or matched—with standardised diaphragm ultrasound procedures, blinded dual-rater analysis, and extended follow-up is necessary to assess durability and real-world impact on exacerbations, health status/quality of life, and diaphragmatic workload.

## Figures and Tables

**Table 1:** Baseline patient characteristics.

Characteristic	N = 21
Age, mean (±SD), years	66.10 (±9.66)
Male gender, N (%)	8 (38.10)
Female gender, N (%)	13 (61.90)
Weight, mean (±SD), kg	77.60 (±13.70)
Height, mean (±SD), cm	169.00 (±6.52)
BMI, median (IQR), kg/m^2^	27.50 (21.00–30.50)
Smokers and ex-smokers, N (%)	7 (33.33)
CRSwNP, N (%)	6 (28.57)
GERD, N (%)	4 (19.05)
Atopy, N (%)	10 (47.62)
On treatment with high-dose ICS/LABA, N (%)	21 (100.00)
T2-high endotype, N (%)	11 (52.38)
T2-low endotype, N (%)	10 (47.62)
GINA treatment step 4, N (%)	6 (28.57)
GINA treatment step 5, N (%)	15 (71.43)
Exacerbations in the last year, mean (±SD)	3.12 (2.56)

Abbreviations: SD, standard deviation; IQR, interquartile range; BMI, body mass index; CRSwNP, chronic rhinosinusitis with nasal polyps; GERD, gastroesophageal reflux disease; ICS, inhaled corticosteroid; LABA, long-acting β_2_-adrenergic agonist; GINA, Global Initiative for Asthma.

## Data Availability

The authors will share all individual participant data collected during the trial, after de-identification, with researchers who provide a methodologically sound proposal.
